# Correction to “Body size‐dependent effects on the distribution patterns of phoretic mite species assemblages on Rhynchophorus ferrugineus (Olivier, 1790)”

**DOI:** 10.1002/ece3.10424

**Published:** 2023-08-10

Matos, I., Silva, D., Oliveira, J., Gonçalves, C., Alves, R., Pereira, N., Catarino, F., Ameixa, O. M. C. C., Sousa, J. A., Rangel, L. F., Santos, M. J., & Ayra‐Pardo, C. (2023). Body size‐dependent effects on the distribution patterns of phoretic mite species assemblages on *Rhynchophorus ferrugineus* (Olivier, 1790). *Ecology and Evolution*, 13, e10338. https://doi.org/10.1002/ece3.10338

In the Acknowledgement Section, the authors missed to mention the “FCT project PTDC‐ASP‐PLA ‐6228‐2020”. Below is the updated Acknowledgement section:

The authors thank the editor and two anonymous reviewers for useful suggestions. This work has received financial support from the Portuguese national funds through FCT—Foundation for Science and Technology within the scope of PTDC‐ASP‐PLA‐6228‐2020, PTDC/BIA‐BMA/6363/2020, UIDB/04423/2020 442, UIDP/04423/2020, UIDP/50017/2020, UIDB/50017/2020, LA/P/0094/2020, by an FCT employment contract CEECIND/03501/2017 (to L.F.R.) and research fellowships PTDCASPPLA62282020‐BI_LIC_2021_019 and 2022_083_BI_ASP‐PLA (to I. M.). O.M.C.C.A. is funded by national funds (OE), through FCT, in the scope of the framework contract foreseen in the numbers 4, 5 and 6 of the article 23, of the Decree‐Law 57/2016, of August 29, changed by Law 57/2017, of July 19. The funders had no role in the design of the study; in the collection, analyses or interpretation of data; in the writing of the manuscript; or in the decision to publish the results.

There is also an error in the legend of Figure 4b. For clarity, the “phoretic mite taxa” are no longer at the top but on the right, and the “body parts of the red palm weevil” are on the left and not at the bottom. The revised legend for Figure 4 should read as follows:

FIGURE 4 Distribution patterns of phoretic deutonymphs on red palm weevil body. (a) Schematic drawing of the six body parts of the red palm weevil examined for the presence of phoretic mites, that is, neck, head–antenna, thorax, legs, abdominal sterna and subelytral space (membranous hind wings + inner elytra surface). (b) Bipartite graph illustrating the interaction between phoretic mite taxa (right) and body parts of the red palm weevil (left; 7 mite taxa × 6 body parts matrix). Interactions between right and left are represented by edges coloured according to the mite taxon involved, with thickness proportional to the number of interactions. (c) Dendogram showing hierarchical clustering of the 7 mite taxa × 6 body parts matrix, performed with the R package pvclust. Values on the branches are approximate unbiased (AU) p‐values (red, left) and bootstrap probability (BP) (green, right), expressed as percentages (Suzuki & Shimodaira, 2006). Clustering method: ward.D (Szekely & Rizzo, 2005); distance: Euclidean.

We apologize for these errors.

